# Tell me who your friends are?! The mediating role of friends’ use in cannabis abuse

**DOI:** 10.47626/2237-6089-2021-0269

**Published:** 2021-04-26

**Authors:** Paulo C. Dias, Sílvia Lopes, José Antonio García del Castillo

**Affiliations:** 1 Universidade Católica Portuguesa Faculdade de Filosofia e Ciências Sociais Centro de Estudos Filosóficos e Humanísticos Braga Portugal Universidade Católica Portuguesa, Faculdade de Filosofia e Ciências Sociais, Centro de Estudos Filosóficos e Humanísticos, Braga, Portugal.; 2 Faculdade de Psicologia Centro de Investigação em Ciência Psicológica Universidade de Lisboa Lisboa Portugal Faculdade de Psicologia, Centro de Investigação em Ciência Psicológica, Universidade de Lisboa, Lisboa, Portugal.; 3 Universidad Miguel Hernández de Elche Elche Alicante Spain Universidad Miguel Hernández de Elche, Elche, Alicante, Spain.

**Keywords:** cannabis abuse, onset age, friends using cannabis, mediation

## Abstract

**Objectives:**

To evaluate the relationship between age at onset of cannabis use and cannabis abuse in a sample of Portuguese cannabis users, testing the mediating role of the number of friends using cannabis and comparing these relationships between male and female subsets.

**Method:**

A sample of 529 Portuguese cannabis users comprising 276 males and 244 females aged from 14 to 21 years completed a sociodemographic questionnaire, the Cannabis Abuse Screening Test (CAST), and answered four questions related to cannabis use. Data were analyzed using the PROCESS macro in SPSS version 26.0.

**Results:**

Age at onset of cannabis use was negatively related to the number of friends using cannabis, while the number of friends using cannabis had a positive relationship with cannabis abuse. As predicted, the number of friends using cannabis seems to have a mediating role in the relationship between cannabis onset age use and cannabis abuse, since the indirect effect was found to be significant. The pattern of the relationships among the variables observed was found to be similar for both male and female subsets of the sample. However, males reported higher cannabis abuse than females.

**Conclusion:**

The results highlight the mediating role of friends’ use of cannabis in the relationship between age at onset of cannabis use and users’ abuse. These results highlight the importance of early intervention in cannabis use. In particular, the pattern of the relationships observed among the studied variables emphasizes the need to monitor and conduct peer training interventions or interventions to promote of social skills.

## Introduction

According to the World Health Organization (WHO),^1^ cannabis is a psychoactive substance of natural origin that exerts an action on the central nervous system and modifies one or more of its functions, perceptions, emotions, attitudes, and behaviours.^1^ It is currently the most widely used illicit substance^2^ and it has been estimated that 3.8% of the global population between 15 to 64 years old used cannabis in the past year, an increase of about 30% between 1998 and 2017. Among Europeans aged from 15 to 34, estimated use is around 15%, rising to 19.2% among those aged 15 to 24.^3^ In Portugal, prevalence of cannabis use has been estimated at 9.7% between the ages of 15 and 74, with 4.5% reporting use in the previous 12 months.^4^ According to a survey of young people participating in the National Defense Day, aged 18 in 2019, prevalence of cannabis use in the last year reached 26.9%. These data allow us to understand the extent of this public health problem. In fact, cannabis is the most used illicit substance, regardless of age, gender, or region of residence, with a prevalence of consumption during the last year that is higher than the European average and still increasing.^4^

Some factors have been associated with cannabis use. It tends to be higher in males,^5-8^ related to parenting practices and parental cannabis use,^9-12^ as well as to friends’ use.^13-15^ Also, ease of access,^8^ in a culture of complacency or permissiveness,^16^ being perceived as harmless^17,18^ and even normalized,^19^ contribute to its growing use among adolescents.

In a developmental period that is well-known for its challenges and changes,^20^ during which the peer group becomes a source of identity and bonding, students desire to “fit in” and substances are used as a strategy to manage their anxiety, particularly regarding schoolwork, sense of academic failure, and lack of social support.^21^ Although some studies have already explored the effect of friends on cannabis use^13-15^ or age at onset of cannabis use on other psychopathological problems^22,23^ and cannabis abuse or other harmful substance use behaviours,^7,24^ there is a lack of evidence relating to the mediating role of friends’ use in the relationship between age at onset of cannabis use and users’ abuse of this substance. The present study explored the relationships between age at onset of cannabis use, number of friends using cannabis, and cannabis abuse, in particular, testing the mediating role of the number of friends using cannabis. In addition, since previous studies suggest a moderating role of certain demographic characteristics,^5-8^ such as when comparing men and women,^5^ the current study explored the relationships among the age at onset of cannabis use, the number of friends using cannabis, and cannabis abuse, comparing male and female subsets. Bearing in mind the conceptual model under study, this paper has the potential to contribute to both literature and practice. First, we explore the mediating role of the number of friends using cannabis, contributing to explain the relationship between the age at onset of cannabis use and cannabis abuse. To the best of our knowledge, this is the first study testing a more complex model by including the number of friends using cannabis as an intervenient variable that contributes to explain the relationship between the age at onset of cannabis use and cannabis abuse. Second, the current study tests whether there are differences between males and females in the relationships between age at onset of cannabis use, number of friends using cannabis, and cannabis abuse. Furthermore, based on the results obtained, we expect to contribute to indicating a set of practices to be implemented in the psychotherapeutic process with cannabis users and peer training or promotion of social skills interventions focused on social groups with a history of substance use.

### Age at onset of cannabis use and cannabis abuse

Several studies in the literature report increasing cannabis use, particularly since the end of the first decade of the millennium,^6,25^ with the largest percentage of first experimentation with cannabis occurring before the age of 20.^26^ According to a systematic review of studies on the prevalence and risks of cannabis use disorder among users, performed by Leung et al.,^27^ one in every eight individuals who use cannabis will develop cannabis dependence, with the greatest risks associated with early initiation and frequent use in the adolescent population. Similar evidence is found in the literature. Adolescents who started using cannabis before 15 years of age were at higher risk of developing drug abuse symptoms by age 28.^24^ This issue assumes particular importance since several studies associate higher risks of cannabis and other drug abuse with early age of onset.^7,26-28^ A proportion of subsequent adverse educational outcomes,^28-30^ and early, frequent, and heavy cannabis use, is strongly associated with cognitive and mental health problems in adulthood.^31^ Moreover, evidence shows that brain impairment is greater in adolescents who use cannabis when compared with adults.^32-34^ Bearing in mind the literature, this study’s first hypothesis was as follows:

Hypothesis 1 (H1): age at onset of cannabis use has a negative relationship with cannabis abuse.

### Indirect effects: a proposal for the mediating role of the number of friends using cannabis

As previously noted, the literature suggests that age at onset of cannabis use is significantly related to cannabis abuse.^27^ Going one step further, the current study suggests that beyond this direct relationship, an indirect relationship can also occur via the number of friends using cannabis.

In the literature, most studies explore the relationship between the number of friends using cannabis and cannabis abuse. Some authors point out the role of socialization and identification with groups that they consider to be similar from the point of view of substance use.^35^ Also, through social learning,^36^ adolescents imitate their high-status peers, leading to involvement in cannabis use.^37^ Particularly with the increasing time children and adolescents spend at schools, this context assumes a determinant role in their behaviors. Authors such as Fletcher et al.^21^ explore how this context influences drug use and find three main motives: students who do not identify with indicators of success find a source of identity and connection in substance use; students who want to feel included in schools that are considered unsafe and the use of drugs facilitating this process; but also substance use might be used as a coping strategy for schoolwork or unhappiness.^21^ Regardless of the motives, several studies have identified friends using cannabis as a predictor of substance use.^15,38^ Moreover, a recent study^14^ with data from the 2011 European School Survey Project on Alcohol and other Drugs (ESPAD),^39^ involving nearly 80,000 15-to-16-year-old students from 25 countries in the European Union plus Norway, found that the most important predictor of substance abuse was the number of friends using substances. Furthermore, youth tend to be very accurate in their perceptions of the frequency of their friends’ substance use.^6,40^ However, friends’ influence on cannabis use might play out in different ways.^41^ Despite some contradictory evidence,^42^ research exploring the influence of age at onset of cannabis use on friendship selection is less common. However, some evidence highlights friend selection based on similar lifetime and cannabis use.^43,44^ Moreover, in a recent longitudinal study following 1,030 boys from 6 to 28 years of age, Rioux et al.^24^ found the effect of onset age of cannabis use in cannabis abuse was mediated by the indirect effect of affiliation with deviant friends. In this context, we assume that the number of friends using cannabis might have an important role, mediating the relationship between age at onset of cannabis use and cannabis abuse. Therefore, a mediating hypothesis was derived as follows:

Hypothesis 2 (H2): the number of friends using cannabis has a mediating effect on the relationship between the age at onset of cannabis use and cannabis abuse.

### Comparing males and females regarding the relationships studied

In the literature, we find consistent data related to higher rates of cannabis use in males than females.^5-8^ This trend is similar for overall substance use and, according to the European Drug Report 2021, cannabis was used by approximately 47.5 million males compared to 30.9 million females in the European Region.^3^ This trend is naturally followed in Portugal, not only in overall cannabis use, but also in moderate to high-risk use in men and women.^4^ Authors of a longitudinal study with male-female sibling pairs spanning more than 20 years explored factors related to substance use disorders.^45^ If males were at higher risk of early-onset alcohol use, the trend for cannabis was the opposite. Females tend to be involved in cannabis use earlier than males. Along the same lines, authors find gender differences in the developmental trajectories of substance use progression between substances.^46^ Not only does the effect of cannabis use onset age seem to be different between men and women,^47,48^ but these differences have also been explored considering the roles of group membership, identity construction, and relations with peers in terms of their influence on cannabis use patterns.^49^ Bearing in mind the findings of these abovementioned previous studies, it is possible to expect males and females to differ in terms of the relationships analyzed in the present study. As such, this study’s third hypothesis was as follows:

Hypothesis 3 (H3): the relationships between age at onset of cannabis use, the number of friends using cannabis, and cannabis abuse differ comparing the male and female subsets.

## Method

### Procedures and sample

The sampling procedure used was a non-probabilistic sample with a convenience sampling approach. The questionnaire was administered in educational settings, at regular and vocational schools in the north and center of the country, using a paper and pencil format. The research team contacted secondary level institutions, presenting the aims and the methodology of the study. After the educational institutions had granted approval and parents’ informed consent had been obtained, teachers were contacted to operationalize the data collection process. Data were collected in the classroom, by a researcher, from all students who presented informed consent signed by their parents or guardians. Individuals’ anonymity, confidentiality, and voluntary participation were guaranteed in the informed consent form, which also presented the study’s main goal. The present study was conducted in line with the Helsinki Declaration and ethical aspects were taken into consideration in development of the study. Additionally, the research was carried out in compliance with institutional and national standards. More precisely, this study was approved and screened for ethical compliance by the ethics board at the research center of the Portuguese university where the research was conducted. This consists of an independent board of researchers belonging to different research fields in the humanities and social sciences. Thus, the board ensures impartial evaluation of the research, avoiding conflicts of interest and prioritizing respect for ethical standards.

A sample of 529 Portuguese cannabis users was extracted from the overall sample of 4,122 participants. The members of this subset were selected based on their answers to ESPAD questions.^39^ We excluded participants who answered that they had never used cannabis over life or in the last 12 months, 30 days, or 7 days. Participants were aged from 14 to 21 years (mean [M] = 16.90; standard deviation [SD] = 1.26) and the majority were male (53.90%). Most participants’ fathers and mothers had attained their 9th year of education (34.2 and 25.8%, respectively) or 12th year of education (44.2 and 50.7%, respectively) and a majority of the sample’s parents were married (59.2%). Both male and female subsets had approximately the same mean age and equivalent demographic characteristics in terms of their parents’ education and marital status. A description of the sample is reported in Table 1.

**Table 1:** Demographics of the sample

	Total sample (n = 529)	Males (n = 276)	Females (n = 244)
Age, mean (SD)	16.90 (1.26)	17.01 (1.26)	16.77 (1.27)
Father’s education (%)			
Illiterate	1.0	1.6	0.4
9 years at school	34.2	34.7	33.6
12 years at school	44.2	45.1	43.2
University degree	14.3	13.0	15.9
Not answered	6.2	5.5	7.0
Mother’s education (%)			
Illiterate	0.5	0.6	0.4
9 years at school	25.8	27.2	24.3
12 years at school	50.7	51.9	49.3
University degree	18.4	15.2	22.1
Not answered	4.6	5.1	4.0
Marital status (%)			
Married	59.2	60.3	58.0
Single	4.7	6.2	2.9
Widowed	4.7	5.2	4.0
Divorced	28.1	25.2	31.5
Other	3.3	3.1	3.6

SD = standard deviation.

### Measures

A sociodemographic questionnaire was used to collect information about the participants and their backgrounds, such as sex, age, and parents’ education and marital status.

The Cannabis Abuse Screening Test (CAST)^50^ is a measure with six items to evaluate the risk associated with the individual’s cannabis use pattern. Answered on a five-point rating scale (from 0 = never to 4 = very often), this screening test was developed based on DSM-IV and presented good validity with a bifactorial model and high reliability (α = 0.81) and is compatible with DSM-V standards.^51^ The total score is summed after recoding the item responses as 0 or 1 to give a total score ranging from 0 to 6.^50^ If participants answer 0 = never or 1 = rarely, their answer is recoded as 0, while the remaining options, 2 = from time to time, 3 = fairly often, or 4 = very often, are recoded as one. Higher scores indicate a higher risk of cannabis abuse, which is classified into four levels, where 0 = no risk, 1 to 2 = low risk, 3 = moderate risk, and 4 to 6 = high risk of cannabis abuse.

Four questions related to cannabis use, over life, in the last 12 months, 30 days, and 7 days, age at onset, and friends’ use were collected from the ESPAD.^39^ These questions are answered on an ordinal scale, graded as 0; 1-2 times; 3-5 times; 6-9 times; 10-19 times; 20-39 times; or 40 times or more.

### Data analysis

The analysis consisted of several steps. First, descriptive statistics (mean [M] and standard deviation [SD]) and intercorrelations between the study variables were calculated with the SPSS 26.0 program. Next, also using the SPSS 26.0 program, the linear regression model was applied to analyze the direct relationship between the age at onset of cannabis use and cannabis abuse. The PROCESS macro in SPSS 26.0 was used to conduct regression analysis and test for the existence of mediation effects. The model used when performing the PROCESS macro was Model 4,^52^ which allows up to 10 mediators to operate in parallel. To test the mediation hypothesis, we used 5,000 bootstrap samples with a 95% bias-corrected bootstrap confidence interval for all indirect effects. Finally, z scores were calculated to compare males and females regarding the relationships among the studied variables (i.e., H3).

## Results

### Descriptive statistics

On average, the participants of the present study were 15 years old (SD = 1.71) when they used cannabis for the first time and they had approximately three friends who use cannabis (SD = 1.03). Regarding the scores recorded for cannabis abuse, the mean value obtained (M = 1.26; SD = 1.68) indicates that, on average, these participants presented a low level of cannabis abuse. Comparison of the male and female subsets of the sample detected no significant differences in age at onset of cannabis use or number of friends using cannabis [t (528) = 1.19, not significant; t (528) = 1.79, not significant]. However, males reported a higher level of cannabis abuse compared to females [M = 1.47, SD = 1.84; M = 0.96, SD = 1.38; respectively; t (528) = -3.88, p < 0.01].

In general, the pattern of correlations observed indicated that age at onset of cannabis use correlates negatively with the number of friends using cannabis (r = -0.20, p < 0.01) and cannabis abuse (r = -0.38, p < 0.01). Furthermore, the number of friends using cannabis was positively related to cannabis abuse (r = 0.34, p < 0.01).

### Hypothesis testing

**Figure 1:**
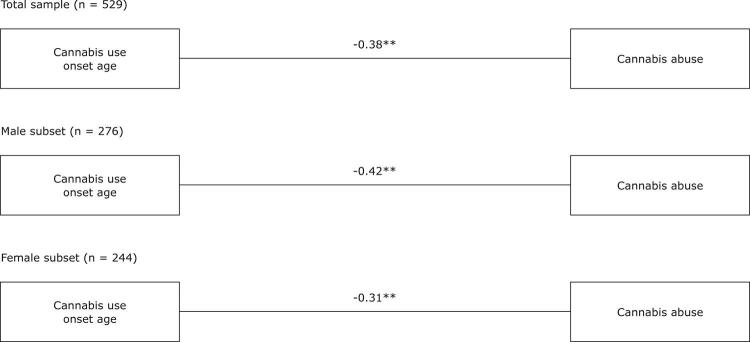
Direct relationship models (before adding the proposed mediating variable). The figure includes the standardized estimates (β). * p < 0.05; ** p < 0.01.

The correlation results provide a general idea of the pattern of the relationships between the constructs. Thus, the next step in data analysis, before mediation testing, consisted of estimating a model that included only the direct relationship between cannabis use onset age and cannabis abuse (Figure 1). This model was tested without the hypothesized mediating variable (i.e., number of friends using cannabis) and a negative relationship was observed for the total sample, and for the male and female subsets analyzed separately (β = -0.38; p < 0.01; β = -0.42; p < 0.01; β = -0.31; p < 0.01; respectively). Thus, H1 was supported by the data.

**Figure 2:**
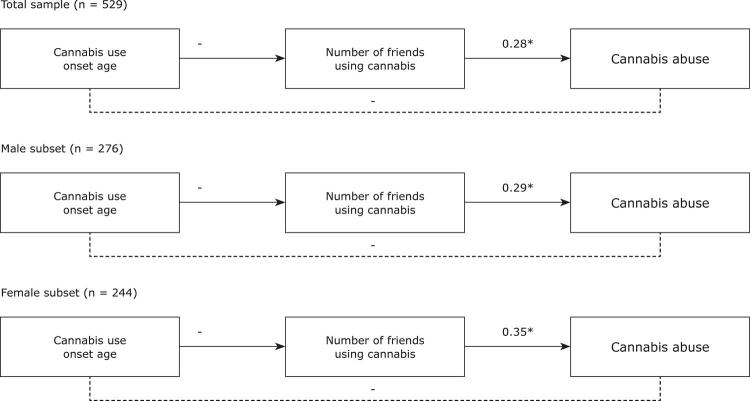
Mediated relationship models (with the proposed mediating variable added). The figure includes the standardized estimates (β). * p < 0.05; ** p < 0.01.

Proceeding with the hypotheses testing, another model was tested with the hypothesized mediating variable (i.e., number of friends using cannabis) included. As can be seen in Figure 2, negative relationships were found between age at onset of cannabis use and the number of friends using cannabis for the total sample and for the male and female subsets analyzed separately (β = -0.20; p < 0.01; β = -0.23; p < 0.01; β = -0.17; p < 0.01; respectively). In turn, positive relationships were found between the number of friends using cannabis and cannabis abuse for the total sample and for the male and female subsets analyzed separately (β = 0.28; p < 0.01; β = 0.29; p < 0.01; β = 0.35; p < 0.01; respectively). Bearing in mind the results presented in Figure 1 for the direct relationship between age at onset of cannabis use and cannabis abuse, it can be observed that this relationship was weakened when the mediator variable was included in the model (Figure 2). Moreover, the indirect effect of age at onset of cannabis use on cannabis abuse via the number of friends using cannabis was found to be significant for the total sample and for the male and female subsets analyzed separately (estimate = -0.06, 95% confidence interval [95%CI] [-0.09 -0.03]; estimate = -0.07, 95%CI [-0.12 -0.03]; estimate = -0.06, 95%CI [-0.11 -0.01]; respectively). Thus, the number of friends using cannabis seems to have a mediating role, contributing to explaining the relationship between age at onset of cannabis use and cannabis abuse, thereby supporting H2.

Z scores were calculated to test whether the relationships between age at onset of cannabis use, the number of friends using cannabis, and cannabis abuse differ if the male and female subsets are compared. Contrary to what had been expected, males and females did not differ significantly in terms of the relationship between the age at onset of cannabis use and the number of friends using cannabis (z = 0.62, not significant), the relationship between the number of friends using cannabis and cannabis abuse (z = -0.08, not significant), or the relationship between age at onset of cannabis use and cannabis abuse (z = 1.47, not significant). Therefore, H3 was not supported.

## Discussion

The main purpose of the present research was to study an unexplored mediating role of the number of friends using cannabis to explain the relationship between age at onset of cannabis use and cannabis users’ abuse. This study also aimed to compare males and females in terms of the relationships between the studied variables. Starting with a descriptive study, we tested three hypotheses: H1 – Age at onset of cannabis use has a negative relationship with cannabis abuse; H2 – The number of friends using cannabis has a mediating effect on the relationship between the age at onset of cannabis use and cannabis abuse. H3 – the relationships between age at onset of cannabis use, the number of friends using cannabis, and cannabis abuse differ comparing the male and female subsets.

Following the existing literature, we found higher cannabis abuse among males^5-8^ and first experience with cannabis at an average age of 15 years. Despite differences in the literature,^32,33^ this average cannabis use onset age tends to be defined as an early-onset age,^34^ associated with higher risks of educational,^28-30^ cognitive, and mental health problems.^31^ This is a cause for deep concern that should be explored in further research and is a finding that underscores the need for preventative interventions. If we consider, as found by Rioux et al.,^24^ that for each year by which onset of cannabis use is delayed, the probability of developing any drug abuse symptoms is reduced by 31%, we understand the urgency of continuing to study and of promoting adjusted interventions.

Previous studies have suggested that age at onset of cannabis use is negatively related to cannabis abuse^24,26-27^ and the current study confirms the predicted relationship. Thus, the older an individuals’ age at onset of cannabis use, the lower their level of cannabis abuse. In other words, individuals who started to use cannabis at an older age seem to report a lower level of cannabis abuse. The pattern of this relationship was found not to differ when male and female subsets of the sample were compared. Also, the number of friends using cannabis was associated with cannabis abuse.^13-15^ As part of socialization or identification with peers with the same lifestyle,^35,37^ these results highlight the need to promote early prevention strategies and not only at the individual level but in school and contexts of living.

More interestingly, our study found support for the role of the number of friends using cannabis in explaining the relationship between the age at onset of cannabis use and users’ abuse. However, we only found support for partial mediation, which seems to indicate that there may be other variables playing a mediating role in explaining the relationship between the age at onset of cannabis use and users’ abuse. Future studies should continue to analyze the mediating role of the number of friends using cannabis in explaining the relationship between the age at onset of cannabis use and users’ abuse but adding some other constructs as mediators, such as behavioral problems^42^ or parental monitoring.^10^ Additionally, since, in the present study, there was no apparent difference in the mediated relationship observed between the male and female subsets of the sample, future studies should continue to analyzing the two samples separately to see if the pattern of the relationships observed remains.

Although not hypothesized, it should be emphasized that a significant difference was found between the male and female subsets in terms of their levels of cannabis abuse. More precisely, males reported higher cannabis abuse than females. This result seems to be in line with what has already been found in previous studies.^5-8^ However, no significant differences were observed between males and females regarding the age at onset of cannabis use or the average number of friends they have who use cannabis.

Although this study has strengths, some limitations should also be noted. First, it is important to highlight that the sample evaluated in this study comprised individuals from only one country (Portugal), which may constrain generalization of these results. Additionally, the current study has a cross-sectional design and so establishment of causal relationships among the studied variables is compromised. It is therefore important that future research examine these relationships over time. However, as Spector noted,^53^ “there seems to be a universal condemnation of the cross-sectional design and at the same time acceptance of the superiority of the longitudinal design in allowing conclusions about temporal precedence and even causality. Often overlooked is that the cross-sectional design can tell us much that is of value and that the longitudinal design is not necessarily superior in providing evidence for causation” (p. 125).

Additionally, the average CAST score suggests a low risk of cannabis abuse in the sample. This might be a limitation of the study, which does not represent a normal distribution, but might reflect the reality of the users in our context. In fact, according to the most recent epidemiological data for Portugal, only 0.3% of the population were at moderate risk of cannabis abuse and 0.4% at high risk.^4^ Considering these issues, future work should also consider other methods of measurement beyond self-report; for example, drug tests including urine, saliva, blood, and perspiration. Despite the previous evidence of the validity of this measure, correlated with cannabis abuse disorders according to DSM-5,^51^ comparison with clinical samples could contribute important additional data to clarify the mediating role of friends’ use in cannabis abuse. Also, new studies considering functional magnetic resonance imaging (fMRI) may allow us to understand the dynamics between onset age and its effects on educational, health, and social outcomes. Replicating these findings with other methods of measurement may be useful for validating the findings of the present study. However, to minimize this limitation, we followed methodological recommendations proposed by Podsakoff et al.,^54^ specifically, guaranteeing the anonymity and confidentiality of the answers and indicating that there were no right or wrong answers.

## Conclusion

The present study allows us to confirm the mediating role of the number of friends using cannabis in the relationship between the age at onset of cannabis use and users’ abuse. The mediation effect found is a very interesting finding, since it allows us to theorize and understand the effect of the variables, with implications for prevention and intervention programs.

In a context of increasing complacency or permissiveness, these findings allow us to understand the crucial role of early intervention, in educational contexts, preventing early exposure of youth to cannabis risk, but also the need to monitor and conduct peer training interventions or promotion of social skills focused on social groups with substance use history. Selective programs might be important in this strategy to delay onset age, decrease the level of cannabis abuse, or motivate treatment. Given the trend of increasing prevalence of cannabis use in European countries and the age of onset, efforts should be strengthened not only to avoid the escalating effect that is to be expected, but also to postpone or prevent experimentation by new users. Besides face-to-face interventions, new strategies using new technologies should also be implemented to address this public health problem.
